# Temporal Changes in Racial Disparities of HIV Linkage to Care from 2013 to 2020: A Statewide Cohort Analysis

**DOI:** 10.1007/s40615-025-02355-3

**Published:** 2025-02-28

**Authors:** Fanghui Shi, Katherine E. Weaver, Chen Zhang, Bankole Olatosi, Jiajia Zhang, Sharon Weissman, Xiaoming Li, Xueying Yang

**Affiliations:** 1 Arnold School of Public Health, South Carolina Smartstate Center for Healthcare Quality, University of South Carolina, 915 Greene Street, Columbia, SC 29208 USA; 2 Department of Health Promotion, Education and Behavior, Arnold School of Public Health, University of South Carolina, 915 Greene Street, Columbia, SC 29208 USA; 3 Department of Psychology, University of South Carolina College of Arts and Sciences, Columbia, SC 29208 USA; 4 Department of Epidemiology and Biostatistics, Arnold School of Public Health, University of South Carolina, Columbia, SC 29208 USA; 5 Department of Health Services Policy and Management, Arnold School of Public Health, University of South Carolina, Columbia, SC 29208 USA; 6 Department of Internal Medicine, School of Medicine, University of South Carolina, Columbia, SC 29208 USA

**Keywords:** HIV, Racial disparity, Linkage to care, South Carolina

## Abstract

**Background:**

Racial disparities have historically existed regarding HIV care outcomes, including linkage to care. This study aims to explore the contribution of contextual features (e.g., socioeconomic and structural environmental factors) to the temporal change of county-level racial disparities in linkage to care.

**Methods:**

This is a statewide population-based retrospective cohort study. The patient-level variables in the South Carolina HIV registry system were used to calculate the aggregated county-level linkage to care percentage. Then, we used four indices to measure racial disparities in the county-level percentage of timely linkage to care, i.e., the Black-White ratio, index of disparity (ID), weighted ID, and Gini coefficient. Linear mixed-effect models were used to estimate the relationship between a variety of contextual features and disparity indexes. The analysis included data from 2013 to 2020, with 2013 as the start year due to the availability of key contextual features and 2020 as the end year based on the most recent HIV registry data available at the time of this study.

**Results:**

Across 46 counties in South Carolina, racial disparity in linkage to care persisted between 2013 and 2020, as indicated by all four indices. When using ID, weighted ID, and Gini as outcomes, counties with lower degrees of racial residential segregation and stronger family structure were at higher risk of racial disparities in linkage to care. For weighted ID only, counties with fewer primary care providers (*β* =  − 4.22; 95% CI, − 7.23 ~ 1.23) had larger racial disparities in linkage to care. Furthermore, for Gini only, counties with higher income inequalities (*β* = 0.01; 95% CI, 0.00 ~ 0.02) had larger racial disparities in linkage to care.

**Conclusion:**

Efforts to address racial disparities should continue, and innovative approaches, specifically those that focus on social and structural factors, should be developed and implemented for populations that have poor HIV outcomes in the USA.

**Supplementary Information:**

The online version contains supplementary material available at 10.1007/s40615-025-02355-3.

## Introduction

Timely linkage to care, defined as having records of HIV viral load or CD4 count measurement within 1 month of initial HIV diagnosis, is crucial for achieving viral suppression and reducing secondary transmission [1]. Linkage to care is the first step in the HIV care continuum, enabling the initiation of antiretroviral therapy (ART), which, when consistently adhered to, can promote viral suppression [2]. Viral suppression not only improves health outcomes for people with HIV (PWH), but also significantly reduces HIV transmission risk [3]. Thus, linkage to care is a critical component in achieving the public health goals of the Ending the HIV Epidemic (EHE) plan, which aims to reduce new HIV infections in the USA by 75% by 2025 and by 90% by 2030 [4–6]. In 2022, the estimated number of people newly diagnosed with HIV among persons aged 13 years and above was 31,800 in the USA and around 800 in South Carolina [7–10]. Of all people newly diagnosed with HIV, the timely linkage to care rate was 82% in the USA, while only 78.1% in South Carolina [7, 8].

Racial inequities are highly pervasive in HIV linkage to care, and understanding underlying social factors that accelerate such disparities is critical for the development of effective HIV prevention and treatment strategies [11]. In 2022, 78.3% of Black/African American people newly diagnosed with HIV in the USA were linked to care within 1 month of HIV diagnosis, which is notably lower than the 82.1% observed among White people newly diagnosed with HIV [12]. These disparities are similarly reflected in South Carolina, in 2022, 76.0% of Black/African American people newly diagnosed with HIV were linked to care, compared to 77.3% of White individuals [13]. HIV-related place-based studies, which focus on examining a specific location and incorporating local history, culture, environment, and community dynamics as key factors of disparities in HIV-related health outcomes, illuminated the significant role of neighborhood characteristics in shaping racial disparities in linkage to care [14].

Racial segregation and the contextual factors (e.g., poverty rates, housing policies, insurance rates) that accompany it can contribute to poor linkage to care in Black/African American communities [15, 16]. For example, individuals living in counties with higher percentages of population with an income below the federal poverty level had lower linkage to care rates [17]. Members of racial and ethnic minorities are often subject to social and economic disadvantages, and poor socioeconomic conditions (i.e., lack of insurance and low income) are associated with delayed linkage to care [18]. Additionally, religious adherence, which is prevalent in South Carolina, may also contribute to racial disparities in linkage to care by influencing health-seeking behaviors and access to care through community norms, stigma, or support networks [19, 20].

Despite existing efforts to examine racial disparities in HIV care outcomes, there are some significant knowledge gaps. First, the current literature’s measurement of the presence and extent of racial disparities in linkage to care was limited to rate differences or rate ratios (such as the black-white rate ratio) [21–23]. Except for these two measurements, the index of disparity (ID, weighted and unweighted version) and Gini coefficient are composite measures that account for disparities across different racial groups and have previously been used to calculate racial disparities in the context of HIV diagnoses but not linkage to care [24]. Different from rate ratio or rate difference, which measures how many times or how many percentages higher (or lower) one group’s rate is compared to the other, ID (weighted and unweighted version) and Gini coefficient reflect the overall disparity between two groups. Experts in quantifying disparities have suggested using multiple measures instead of relying on any single measure to provide a comprehensive view of racial disparities in disease burden. Using multiple indexes helps capture different aspects of disparities, guiding more effective and equitable public health interventions [25]. Second, previous literature that has examined racial disparities in linkage to care rates or contextual predictors of linkage to care has not used data collected over multiple years [6, 18, 26, 27]. Longitudinal collection in this manner will allow for examining racial disparity patterns over time. Third, there is little geographic variability within existing research as most data were collected from urban areas. There is a need to explore neighborhood factors on a larger scale in more rural areas, such as South Carolina.

To address these knowledge gaps, the current study aims to use statewide longitudinal electronic health records (EHR) data to (1) examine the spatiotemporal variation of racial disparities in linkage to care from 2013 to 2020 across counties in South Carolina and (2) explore the contribution of contextual features (e.g., socioeconomic and structural environmental factors) to the temporal change of county-level racial disparities of linkage to care.

## Methods

### Data Sources and Linkage

All people newly diagnosed with HIV (≥ 18 years) in South Carolina between 2013 and 2020 were included in the study population. The analysis included data from 2013 to 2020, with 2013 as the start year due to the availability of key contextual features and 2020 as the end year based on the most recent HIV registry data available at the time of this study. The enhanced HIV/AID reporting system from the South Carolina Department of Public Health (DPH) includes CD4 counts and viral load for all individuals. These serologic markers assisted in the calculation of the county-level timely linkage to care percentage in accordance with the definition from the Centers for Disease Control and Prevention (CDC). Multiple public databases, including the American Community Survey (ACS) County Health Rankings & Roadmaps, the Federal Information Processing Standards (FIPS), and the United States Congress Joint Economic Committee, provided information for county-level variables. The ACS 5-year estimates were selected to increase statistical reliability for data collection of small population groups across the counties [28]. The institutional review boards at the University of South Carolina and relevant South Carolina state agencies approved the study protocol (IRB #: Pro00121718).

### Racial Disparities in County-Level Percentage of Timely Linkage to Care

People newly diagnosed with HIV with at least one record of HIV viral load or CD4 cell count measurement within 1 month of being diagnosed with HIV were considered “timely linkage to care.” The county-level percentage of timely linkage to care was calculated annually as the number of people newly diagnosed with HIV categorized as “timely linkage to care” divided by the total number of people newly diagnosed with HIV in the specified calendar year. Four indices measured racial disparities in the county-level timely linkage to care percentages—the Black to White ratio, ID, the Weighted version of ID, and the Gini coefficient.

The Black-White ratio was calculated as the percentage of timely linkage to care among Black/African American people newly diagnosed with HIV divided by the percentage of timely linkage to care among White people newly diagnosed with HIV. A Black-White ratio greater than 1 indicates the linkage to care rate is higher for Black individuals compared to White individuals, highlighting a specific disparity between these two groups. The ID is the average of absolute differences between Black/African American and White people newly diagnosed with HIV linkage to care rates divided by the linkage to care rate for all people newly diagnosed with HIV in South Carolina. The ID is expressed as a percentage using the following formula:

$$\mathrm{ID}= 100\times (\frac{{\sum }_{i=1}^{2}\left|{\mathrm{Rate}}_{i}-{\mathrm{Rate}}_{\mathrm{overall}}\right|}{2})/{\mathrm{Rate}}_{\mathrm{overall}}$$

In this case, *i* is the racial group, the rate indicates the percentage of timely linkage to care in the given group, and the overall parentage of timely linkage to care across two racial/ethnic groups combined. Higher values of ID indicate greater variability and overall disparity. The weighted ID uses a similar formula, with the population-weighted average of the absolute difference in linkage to care rates between race groups and the linkage to care rate for the total people newly diagnosed with HIV population in South Carolina. Weighted ID gives more importance to disparities affecting larger groups than to ID, and higher values indicate greater disparities.

$$\text{Weighted ID}= 100\times (\frac{{\sum }_{i=1}^{2}\left|{\mathrm{Rate}}_{i}-{\mathrm{Rate}}_{\mathrm{overall}}\right|\times {\mathrm{Population}}_{\mathrm{i}}}{{\mathrm{Population}}_{\mathrm{overall}}})/{\mathrm{Rate}}_{\mathrm{overall}}$$

The Gini coefficient has previously been used to measure income inequality in the context of HIV diagnosis and sexually transmitted infection (STI) prevention. Black/African American people newly diagnosed with HIV and White people newly diagnosed with HIV were ranked one and two according to linkage to care rates. In this case, the group ranked one had the lower timely linkage to the care rate. The Gini coefficient was calculated using the following formula:

$$\mathrm{Gini}=1- {\sum }_{i=1}^{i=2}({Y}_{i}+ {Y}_{i-1})({X}_{i}- {X}_{i-1})$$

where *Y*_*i*_ and *X*_*i*_ are the cumulative percentage of timely linkage to care and the cumulative percentage of the number of people newly diagnosed with HIV, respectively, accounted for by the Black/African American group and the White group, and *X*_0_ and *Y*_0_ are zero. The Gini coefficient summarized overall inequality across racial groups, and higher values suggest a more unequal distribution of linkage to care.

### County-Level Variables

A total of 24 county-level variables were obtained from multiple publicly available datasets. These variables were further broken up into five categories, including racial residential segregation indices (e.g., Black/White dissimilarity index, isolation index, delta, and spatial proximity), social capital indices (e.g., family structure, community health, institution health, and collective efficiency), social vulnerability sub-indices (e.g., socioeconomic status, household characteristics and disability, minority status and language, and housing type and transportation), healthcare resources and health behavior (e.g., the number of primary care providers, the number of Ryan White HIV centers, the number of mental health centers, smoking%, drinking%, and disability%), and other characteristics (e.g., male%, vacant houses%, unemployed%, uninsured%, Gini index for income inequality, and the percentage of persons with religious adherence). All variables and their definitions can be found in Supplemental Table 1. All 46 counties in South Carolina were further grouped based on their public health regions in South Carolina: Upstate, Midlands, PeeDee, and Lowcountry areas [8].

### Statistical Analysis

First, descriptive statistics, including 25th percentile, median, 75th percentile, and interquartile range (IQR), were utilized to describe the distribution of county-level characteristics across 46 counties in South Carolina in 2020. Second, line plots with linear regression analyses were employed to illustrate the temporal trend of four indices measuring racial disparities in the county-level timely linkage to care percentages from 2013 to 2020 in South Carolina. Third, three linear mixed-effects models were conducted to examine the association of county-level characteristics with three racial disparities in county-level timely linkage to care indices separately, including ID, weighted ID, and Gini coefficient. The Black to White ratio was not included as an outcome in the analysis because the Black to White ratio does not reflect racial disparities in a linear manner, and linear mixed effects modules could oversimplify the complex non-linear relationship between county-level characteristics and racial disparities. The county FIPs code was set as the random effect in the models to adjust for the repeat measures of each county. All analyses were performed in R statistical software version 4.1.2.

## Results

### Descriptive Statistics

**Fig. 1:**
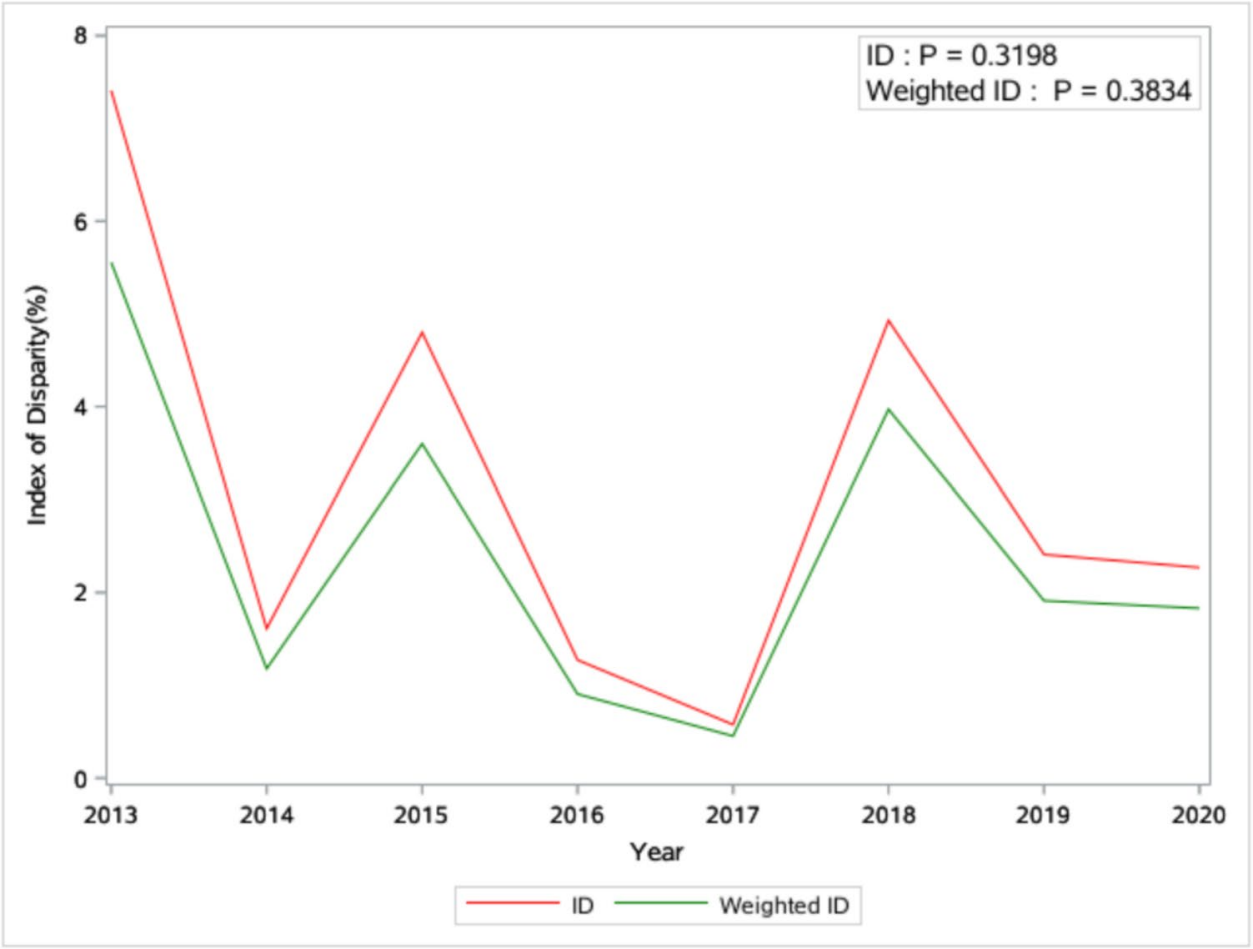
Temporal trend of index of disparity (ID) and weighted version of the index of disparity (weighted ID) from 2013 to 2020 in South Carolina: linear regression analysis

**Fig. 2:**
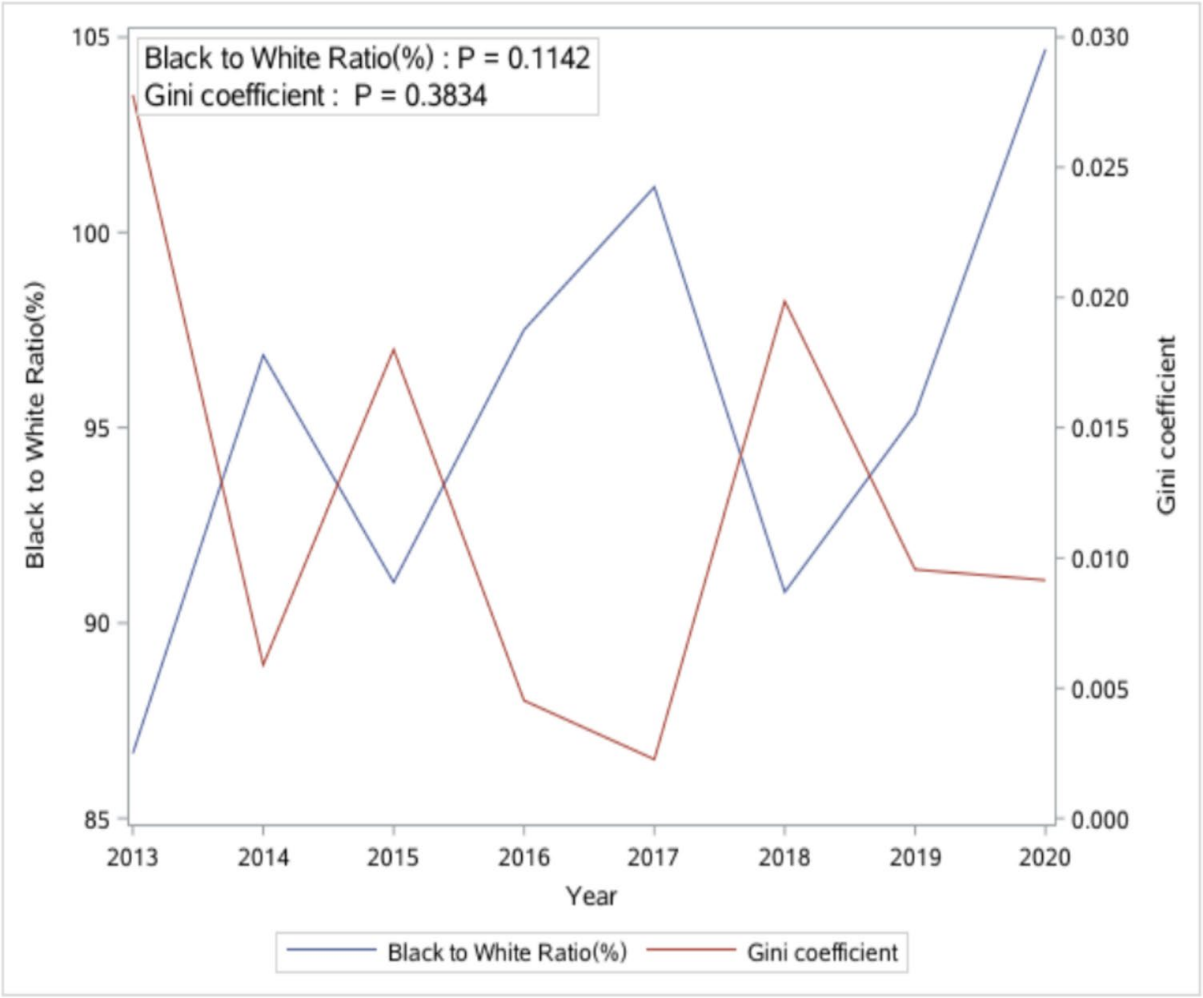
Temporal trend of Black to White ratio and Gini coefficient of linkage to care from 2013 to 2020 in South Carolina: linear regression analysis

Between 2013 and 2020, there were no significant increasing or decreasing trends for all four measurements of racial disparity in linkage to care (p-values are all larger than 0.05) (Figs. 1 and 2). Table 1 shows the distribution of descriptive statistics for county-level variables across the state in 2020. The percentage of the male population and vacant homes stood at a median of 48.43% and 17.08%, respectively. The median percentages for unemployment and uninsured were 6.33% and 10.38%. Additionally, 53.50% (interquartile range [IQR] = 18) of residents were adherent to their religion. On average, each county had just one Ryan White HIV center, 29 primary care providers, and three centers offering mental health services. Additionally, the median (IQR) percentages were 19.00% (3.00%) for smoking, 16.00% (2.00%) for drinking, and 16.20% (4.34%) for disability, respectively.

**Table 1:** Descriptive statistics for county-level variables^a^

	25th percentile	Median	75th percentile	IQR
Racial residential segregation				
Black/White dissimilarity index	25.00	30.50	38.00	13.00
Isolation index	0.41	0.54	0.61	0.19
Delta	0.31	0.46	0.56	0.24
Spatial proximity	0.93	0.95	0.97	0.04
Social capital indices				
Family structure	− 1.81	− 1.16	− 0.51	1.30
Community health	− 0.79	− 0.55	− 0.31	0.48
Institution health	− 0.05	0.25	0.38	0.43
Collective efficiency	122.00	216.50	353.00	231.00
Socio vulnerability indices (SVI)				
SVI_Socioeconomic status	0.60	0.83	0.89	0.29
SVI_Household characteristics and disability	0.42	0.68	0.84	0.42
SVI_Minority status and language	0.60	0.71	0.79	0.19
SVI_Housing type and transportation	0.50	0.73	0.92	0.42
Healthcare resources and health behavior				
Primary care providers	8.00	29.00	74.00	66.00
Ryan White HIV centers	1.00	1.00	3.00	2.00
Mental health centers	3.00	3.00	4.00	1.00
Smoking (%)	17.00	19.00	20.00	3.00
Drinking (%)	15.00	16.00	17.00	2.00
Disability %	13.97	16.20	18.31	4.34
Other characteristics				
Male (%)	48.10	48.43	49.46	1.36
Vacant houses (%)	12.04	17.08	22.18	10.14
Unemployed (%)	5.03	6.33	7.31	2.28
Uninsured %	9.71	10.38	11.49	1.78
Gini index	0.45	0.47	0.48	0.03
Religious adherence (%)	41.00	53.50	59.00	18.00

*IQR* interquartile range

^a^25th percentile, median, 75th percentile, and IQR were only shown for county-level variables in the year 2020 considering limited table space

### The Association of County-Level Characteristics with Racial Disparities in Timely Linkage to Care

All associations in Table 2 are adjusted coefficients derived from three linear mixed-effects models that controlled for all predefined county-level characteristics. Counties with lower degrees of racial residential segregation exhibited a higher risk of racial disparities in linkage to care. This association was consistent across all three measures of racial disparities in linkage to care, including the ID (*β* =  − 7.03; 95% CI, − 9.98 to approximately − 0.67), the weighted ID (*β* =  − 6.28; 95% CI, − 9.86 to approximately − 2.19), and the Gini coefficient (*β* =  − 0.03; 95% CI, − 0.05 to approximately − 0.02). Additionally, counties with stronger family structures demonstrated greater racial disparities in linkage to care, as evidenced by all three measures: the ID (*β* = 7.96; 95% CI, 3.14 ~ 12.9), the weighted ID (*β* = 7.80; 95% CI, 3.73 ~ 11.76), and the Gini coefficient (*β* = 0.03; 95% CI, 0.01 to approximately − 0.04). Furthermore. for weighted ID only, counties with fewer primary care providers (*β* =  − 4.22; 95% CI, − 7.23 to approximately 1.23) had larger racial disparities in linkage to care. For the Gini coefficient only, counties with higher income inequalities (*β* = 0.01; 95% CI, 0.00 ~ 0.02) had larger racial disparities in linkage to care. Some other county-level characteristics, such as the percentage of religious adherence and the percentage of uninsured, were not significantly related to racial disparities in linkage to care, measured by all three indices (e.g., index of disparity, weighted index of disparity, and Gini coefficient).

**Table 2:** Association between county-level contextual factors and racial disparities in linkage to care

	Index of disparity		Weighted index of disparity		Gini coefficient	
	Beta (95%CI)	*p*-value	Beta (95% CI)	*p*-value	Beta (95% CI)	*p*-value
Racial residential segregation						
Black/White dissimilarity index	0.79 (− 3.22, 3.54)	0.717	− 0.12 (− 2.98, 2.59)	0.939	0 (− 0.01, 0.02)	0.567
Isolation index	− 3.19 (− 8.74, 1.52)	0.354	− 3.49 (− 7.7, 0.75)	0.163	–-	–-
Delta	− 7.03 (− 9.98, − 0.67)	0.024	− 6.28 (− 9.86, − 2.19)	0.009	− 0.03 (− 0.05, − 0.02)	0.003
Spatial proximity	− 0.57 (− 3.18, 1.9)	0.705	− 0.63 (− 2.72, 1.45)	0.588	− 0.01 (− 0.02, 0)	0.271
Social capital indices						
Family structure	7.96 (3.14, 12.9)	0.023	7.8 (3.73, 11.76)	0.004	0.03 (0.01, 0.04)	0.005
Community health	− 1.73 (− 4.49, 1.03)	0.328	− 2.11 (− 4.36, 0.17)	0.107	− 0.01 (− 0.02, 0)	0.194
Institution health	− 1.88 (− 4.45, 1.85)	0.37	− 1.25 (− 3.79, 1.4)	0.403	0 (− 0.02, 0.01)	0.444
Collective efficiency	− 1.36 (− 4.11, 1.56)	0.459	− 1.49 (− 3.77, 0.9)	0.266	0 (− 0.01, 0.01)	0.564
Socio vulnerability indices (SVI)						
SVI_Socioeconomic status	− 2.07 (− 6.87, 4.81)	0.566	− 2.46 (− 7.15, 2.47)	0.364	–-	–-
SVI_Household characteristics and disability	1.92 (− 3.24, 6.26)	0.505	0.77 (− 3.17, 4.63)	0.722	–-	–-
SVI_Minority status and language	0.27 (− 2.81, 3.14)	0.889	0.31 (− 2.14, 2.76)	0.821	0 (− 0.01, 0.02)	0.53
SVI_Housing type and transportation	0.01 (− 2.03, 1.94)	0.991	− 0.1 (− 1.77, 1.5)	0.907	0 (− 0.01, 0.01)	0.918
Healthcare resources and health behavior						
Primary care providers	− 4.17 (− 8.22, − 0.95)	0.096	− 4.22 (− 7.23, − 1.23)	0.025	− 0.02 (− 0.03, 0)	0.043
Ryan White HIV centers	0.21 (− 2.29, 3.32)	0.914	− 0.37 (− 2.64, 1.98)	0.783	0 (− 0.01, 0.01)	0.515
Mental health centers	0.37 (− 2.88, 3.15)	0.855	0.01 (− 2.48, 2.47)	0.994	0 (− 0.01, 0.01)	0.706
Smoking (%)	0.24 (− 2.88, 2.03)	0.865	0.84 (− 1.28, 2.76)	0.448	0 (− 0.01, 0.01)	0.635
Drinking (%)	− 0.45 (− 3.05, 2.76)	0.783	0.01 (− 2.39, 2.4)	0.997	0 (− 0.01, 0.01)	0.832
Disability (%)	− 3.05 (− 7.21, 0.78)	0.216	− 2.78 (− 6.06, 0.49)	0.137	− 0.01 (− 0.03, 0)	0.116
Other characteristics						
Male (%)	− 1.23 (− 4.42, 3.8)	0.641	− 0.06 (− 3.28, 3.48)	0.975	0 (− 0.01, 0.02)	0.986
Vacant houses (%)	0.83 (− 1.93, 3.47)	0.652	1.04 (− 1.2, 3.25)	0.423	0 (− 0.01, 0.01)	0.659
Unemployed (%)	0.64 (− 3.26, 4.3)	0.775	0.43 (− 2.69, 3.52)	0.802	0 (− 0.01, 0.02)	0.805
Uninsured %	1.09 (− 1.43, 5.47)	0.59	0.57 (− 2.09, 3.57)	0.719	0 (− 0.01, 0.02)	0.769
Gini index	2.54 (− 0.36, 5.87)	0.165	2.75 (0.24, 5.35)	0.055	0.01 (0, 0.02)	0.042
Religious adherence (%)	− 0.78 (− 3.23, 2.73)	0.705	− 1.05 (− 3.37, 1.53)	0.467	0 (− 0.01, 0.01)	0.639
Region						
MIDLANDS	6.28 (− 1.74, 11.54)	0.18	5.63 (− 0.02, 10.91)	0.093	0.02 (− 0.01, 0.04)	0.193
PEE DEE	− 1.33 (− 6.89, 5.3)	0.745	− 0.28 (− 5.26, 4.77)	0.921	− 0.01 (− 0.03, 0.01)	0.401
UPSTATE	6.09 (− 5.6, 15.97)	0.404	9.17 (− 0.06, 17.69)	0.088	0.02 (− 0.02, 0.04)	0.348

–-, factors omitted during forward selection

## Discussion

This study is one of the first attempts to explore racial disparities in linkage to care using multiple measurements and a statewide longitudinal dataset. The results reveal persistent racial disparities in timely linkage to care in South Carolina from 2013 to 2020. Counties characterized by greater income inequality and limited access to primary care providers exhibited more pronounced racial disparities in linkage to care, as reflected in at least one of the three racial disparity indices. Moreover, higher racial disparities in linkage to care were associated with counties with lower levels of racial residential segregation (as measured by the delta index) and increased social capital in the form of family structure, as reflected in all three racial disparity measurements. Racial disparities in linkage to care in South Carolina are influenced by a complex interplay of socioeconomic, healthcare access, and community factors, and there is a need for targeted interventions to address both structural and community-level determinants in reducing these disparities.

Increased income inequality within a country was associated with an increased risk of racial disparities in linkage to care. Previous research has identified a connection between neighborhood-level income inequality and poorer HIV-related outcomes, and the current findings may further illuminate the role of race in this relationship [14, 29, 30]. For counties with high-income inequalities, racial minorities may be disproportionately concentrated in communities with limited healthcare infrastructure and service [31]. These disparities can lead to gaps in timely linkage to care between racial groups, and the findings regarding the association between fewer primary care providers and larger racial disparities in the current study further prove this hypothesis. Economically disadvantaged communities may also face additional barriers to the linkage to care, such as transportation issues, employment constraints, and lack of health insurance [30, 32, 33]. These barriers may be more pronounced in counties with higher levels of income inequality and further amplifying racial disparities in linkage to care.

Our study found that counties with higher social capital, indicated by stronger family unity (traditional family structures) and lower disability prevalence, are associated with a higher risk of racial disparities in linkage to care. While some dimensions of community-level social capital can be protective, other dimensions are adversely related to health outcomes. For example, Ransome et al. reported that higher neighborhood-level social participation is associated with a higher risk of HIV diagnosis and less linkage to HIV care [34]. One possible explanation for the association between traditional family structure and greater racial disparities in linkage to care is that stronger family unity in certain communities may reinforce HIV-related stigma, creating barriers to disclosure and care-seeking [35]. Black/African American individuals in households with traditional family structures may experience heightened stigma, further exacerbating racial disparities in linkage to care outcomes [35]. Additionally, counties with lower visible social vulnerability may lack targeted public health interventions, further limiting timely linkage to care for racial minorities. These findings highlight the significance of household characteristics and composition in shaping health outcomes across racial groups, emphasizing the need to integrate these factors into linkage to care interventions.

Our finding of lower levels of racial disparity in linkage to care in more racially segregated areas was counterintuitive. It failed to align with previous studies, which found lower percentages of linkage to care in these communities [31]. Although counterintuitive, this finding may reflect targeted public health intervention and enhanced resource allocation in racially segregated communities. Prior studies suggest that communities with greater social vulnerability, partly reflected by minority status and language, usually receive increased funding for public health programs, which may help mitigate health disparities [36]. For example, after exploring HIV testing data from 60 state and local health departments and 119 community-based organizations during 2020–2022, CDC reported that 85.8% of the 4.9 million tests were conducted in high social vulnerability counties, indicating CDC-funded HIV testing programs reach the most vulnerable communities [36]. Furthermore, stronger social or familial networks in racially segregated areas may play a protective role in healthcare access and foster better support for seeking and engaging in care among Black/African American people newly diagnosed with HIV [37]. Variations in segregation measures could also influence the findings and explain the discrepancies between different studies. These hypotheses emphasize the complexity of the relationship between residential segregation and racial disparities in linkage to care, suggesting the need for further explorations to understand the underlying mechanisms of these seemingly contradictory patterns.

Interventions aimed at decreasing racial disparities in linkage to care at the county level need to be culturally sensitive and consider contextual characteristics, such as income inequality and family structure. In areas with higher income inequality, integrating social services, such as financial support, transportation, and housing, into the HIV care model may help mitigate barriers to timely linkage to care [38]. Additionally, recognizing the role of family and social networks in healthcare decisions is crucial. Interventions could include counseling or group programs to empower families to understand HIV care needs and improve family support [39]. Moreover, healthcare providers should receive training on cultural sensitivity to better understand the unique barriers faced by racial/ethnic minority groups, including recognizing the historical mistrust of healthcare systems within communities with higher proportions of these populations [40].

This study has several limitations. First, some counties reported just a few new HIV cases each year, which limits the statistical power of accurately identifying predictors in that area. Second, this study also did not explore the impact of some individual-level psychosocial variables (e.g., HIV-related stigma and mental health needs) on racial disparities in linkage to care rates due to data availability. Additionally, while household composition and structure were found to be associated with the ID and Weighted ID, respectively, this study did not explore data on relationships and connectedness within the household. Similarly, there was limited data on institutional-level practices and characteristics (e.g., average wait time, provider demographics) that may influence racial disparities in linkage to care. Future studies should aim to include other individual, interpersonal, and institutional variables to identify barriers to linkage to care, as this can help with intervention and policy development. Third, since this study extracted county-level data, there is the threat of a modifiable areal unit problem (MAUP). MAUP refers to the problem of potentially inconsistent results when aggregating data based on different geographical boundary definitions, as many geographical units have artificially defined boundaries [41]. Therefore, caution is needed when generalizing findings of this study to other geographic levels or more granular units (e.g., census tracts). Additionally, South Carolina has not adopted Medicaid expansion, which could substantially affect healthcare access for low-income individuals. As a result, the findings may not be generalized to states that have implemented Medicaid expansion. Fourth, this study lacks longitudinal data covering the COVID-19 pandemic and its aftermath, limiting the ability to assess its full and long-term impact [42, 43]. Future research is warranted to examine racial disparities in linkage to care throughout and following the pandemic period.

## Conclusion

Despite national initiatives to increase the number of people newly diagnosed with HIV linked to care, such as the “Ending the HIV Epidemic” plan, it is evident that individuals from marginalized racial identities may still face significant barriers to receiving timely care. The results of this study underscore the need for interventions that not only focus on improving linkage to care but also address the broader social and structural factors that disproportionately affect racial minorities. Our findings suggest that interventions to decrease racial disparities should be culturally sensitive and consider specific contextual factors. Community composition, family structure, and income inequality are among the key contextual factors that can shape healthcare access and outcomes. Policymakers, healthcare providers, and community organizations need to develop and implement multifaceted approaches that address these factors. For example, integrating social services and community education can reduce stigma and alleviate some existing barriers to timely linkage to care. Additionally, promoting policies that address persistent socioeconomic disparities within healthcare systems is crucial for care access. Future studies should further investigate the complex relationship between different contextual factors and racial disparities in healthcare access, aiming to identify specific mechanisms through which these factors influence linkage to care outcomes.

## Supplementary Information

Below is the link to the electronic supplementary material.

Supplementary file1 (DOCX 17 KB)

## Data Availability

The authors are prohibited from making individual-level data available publicly due to provisions in our data use agreements with state agencies/data providers, institutional policy, and ethical requirements. To facilitate research, we make access to such data available via approved data access requests through the data owners. The data is unavailable externally or for re-release due to prohibitions in data use agreements with our state agencies or other data providers. Institutional policies stipulate that all external requests for data access require collaboration with an (author’s affiliation) researcher. For more information or to make a request, please contact (Bankole Olatosi, PhD): Olatosi@mailbox.sc.edu. The underlying analytical codes are available from the authors on request.
