# Supplementary material for: Temporal Changes in Racial Disparities of HIV Linkage to Care from 2013 to 2020: A Statewide Cohort Analysis
Source: J Racial Ethn Health Disparities. 2025 Feb 28;13(2):1572–81. doi: 10.1007/s40615-025-02355-3 (PMC12966212; doi:10.1007/s40615-025-02355-3)
Supplement: Supplementary file 1 — Supplementary file1 (DOCX 17 KB) [file 40615_2025_2355_MOESM1_ESM.docx]

| **Supplemental Table 1. The detailed definition for each county-level variable** | |
| --- | --- |
| Variables | Definitions |
| **Racial residential segregation** |  |
| Black/White dissimilarity index | The percentage of either Black or White residents that would have to move to different geographic areas to produce a distribution that matches that of the larger area |
| Isolation index | The probability that the Black group member would come into contact with another Black group member |
| Delta | The relative amount of physical space occupied by the Black group |
| Spatial proximity | The extent to which neighborhoods inhabited by Black members adjoin one another |
| **Social capital indices** |  |
| Family structure | The share of births that are to unwed mothers, children living in single-parent families, and women aged 35-44 who are married |
| Community health | Non-religious non-profits per capita, congregations per capita, and the informal civil society subindex |
| Institution health | Presidential voting rate, census response rate, and confidence subindex |
| Collective efficiency | Violent crimes per 100,000 people |
| **Socio vulnerability indices (SVI)** |  |
| SVI_Socioeconomic status | Below 150% poverty, unemployed, housing cost burden, no high school diploma, no health insurance |
| SVI_Household characteristics and disability | Aged 65 or older, aged 17 or younger, civilian with a disability, single-parent households, English language proficiency |
| SVI_Minority status and language | Hispanic or Latino (of any race); Black and African American, Not Hispanic or Latino; American Indian and Alaska Native, Not Hispanic or Latino; Asian, Not Hispanic or Latino; Native Hawaiian and Other Pacific Islander, Not Hispanic or Latino; Two or More Races, Not Hispanic or Latino; Other Races, Not Hispanic or Latino |
| SVI_Housing type and transportation | Multi-unit structures, mobile homes, crowding, no vehicle, group quarters |
| **Health Care Resources and health behavior** |  |
| primary care providers | Number of primary care providers per 100,000 population |
| Ryan White HIV centers | Number of Ryan White HIV centers per newly diagnosed HIV cases each year within 25 miles radius |
| Mental health centers | Number of mental health centers per newly diagnosed HIV cases each year within 25 miles radius |
| Smoking (%) | Percent of adults who are current smokers |
| Drinking (%) | Percent of adults reporting binge or heavy drinking |
| Disability % | Percentage of adults with a disability (between ages 18 and 64) |
| **Other characteristics** |  |
| Male (%) | Percent of male persons |
| Vacant houses (%) | Percentage of housing units vacant |
| Unemployed (%) | Percent of 16 years and older persons who are unemployed |
| Uninsured % | Percent of occupied housing units without access to a vehicle |
| Gini index | Income inequality represented by statistical measure of income dispersion |
| Religious adherence (%) | Percent of persons with religious adherence |
